# Registered report protocol: Perceptual effects of Arabic grammatical gender on occupational expectations in a gamified speech production task

**DOI:** 10.1371/journal.pone.0292936

**Published:** 2023-10-20

**Authors:** Farida Soliman, Linnaea Stockall, Devyani Sharma

**Affiliations:** Department of Linguistics, Queen Mary University of London, London, United Kingdom; The University of Lahore, PAKISTAN

## Abstract

The default use of masculine morphology to refer to all genders in Arabic-speaking countries is largely unquestioned and widely accepted. However, research on masculine generic morphology in other gender-marked languages has shown that this can create an over-representation of men and a male-bias in perception. Given the extensive use of default masculine grammatical gender in the context of job recruitment, education, and formal communication where women are typically underrepresented and men overrepresented, this widely accepted notion needs to be investigated. The primary aim of this research is to understand how grammatical gender in Arabic mediates occupational expectations based on the language currently used in job recruitment in Arabic speaking countries. Specifically, the study explores how the use of default masculine grammatical gender can create a male-bias in perception. The secondary aim of this research is to test whether gender-inclusive language can reduce this male-bias in perception and instead increase the accessibility, activation, and retrieval of exemplars related to other gender minorities (i.e., reduce male-bias in perception). This is achieved through a novel prompted speech production experiment, based on an adaptation of the popular board game ’Taboo’ where participants are asked to describe role nouns presented (e.g., *doctor* or *nurse*) in different language conditions.

## Introduction

The default use of masculine morphology to refer to non-male entities, often referred to as the ’ambiguous masculine’ or ’masculine generic’, is common amongst both natural and grammatical gender languages [1]. Its use is relatively widespread and can be seen across various language families, including Romance languages (Spanish, French, and Italian), Germanic languages (Dutch, German, and English), Semitic languages (Arabic and Hebrew), and many more. It is often used to refer to someone with unspecified gender (i.e., where there is ambiguity or a need to refer to a person whose gender is unknown) or to refer to a mixed-gender group. Further, its historic use (cross-linguistically) was largely seen as unproblematic and often used for efficiency [2].

Recently however, there has been a surge in the literature exploring the implications of the use of masculine generics on the representation and perception of women in gender-marked languages such as French, German, Dutch and Italian. These studies have provided ample evidence that grammatical gender plays a major role in mediating gender expectations and representation [3–5]. Specifically, these studies have consistently shown that the generic use of masculine grammatical gender results in an over-representation of men and under-representation of women in perception. Studies have also shown that the effect of grammatical gender on perception is strong enough to exhibit ‘carryover effects’ into another language [6–10].

Thus far this research has been relatively limited to ‘Western’ contexts. This study aims to extend this research on grammatical gender and to test whether masculine generics in other language families, specifically Arabic, behave similarly to Indo-European languages. Arabic is one of the most spoken languages around the world, and yet research on the processing of Arabic grammatical gender remains relatively understudied. Additionally, the use of masculine generics in Arabic speaking countries, especially in the context of job recruitment and formal settings, is largely unquestioned and widely accepted as equally representative of all genders. However, a recent study on the language used in Egyptian job recruitment and advertising showed an overuse and inconsistent use of masculine generics, where stereotypically male-dominated industries used masculine generics and stereotypically female-dominated jobs were advertised in feminine grammatical gender regardless of the gender targeted for the role [11]. Additionally, the study showed that jobs were advertised in both singular (majority) and plural masculine generics, and so a key aspect of this investigation is to determine whether masculine generics in singular or plural are interpreted more or less strongly as male-referring. Therefore, the overall aim of this study is to explore how the presence or absence of grammatical gender affects gender representation in perception.

Specifically, the primary objective of this study is to investigate the role that morphological gender marking plays in either entrenching or perhaps mitigating these segregated and polarised recruitment patterns. To do so, we explore the following questions:

1. How does grammatical gender mediate referent expectations in the presence of competing social information?
  a. Do singular masculine generics create a male bias in perception, and are these biases different in singular and plural masculine generics?
  b. Do participants rely more on stereotypical social information or grammatical information to inform their occupational expectations?
  c. If the social and grammatical cues conflict, do participants exhibit processing costs? Or is there a processing benefit to having congruent social and grammatical cues? And are these benefits or costs consistent across different gender and cue-type combinations?

Additionally, a secondary objective of the study is to develop an understanding as to what kind of language is more likely to promote the retrieval and use of feminine-inflected words and provide a more gender-inclusive interpretation of language. Here we shift our focus to explore the effect of gender-inclusive and gender-neutral language on occupational expectations. To do so, we experimentally test the potential value of using gender-inclusive language as an alternative to default masculine generics to increase women’s representation in cognition by exploring the following questions:

2. Can gender-fair language mitigate gendered expectations and increase the representation of gender minorities?
3. Is gender-neutral language interpreted as such? Specifically, do social gender biases and stereotypes persist, in English, despite the absence of grammatical gender marking?

The third and final aim is to explore some of these effects from a bilingual perspective. Here the study explores whether a subsample of Egyptian bilingual participants exhibit any grammatical gender carry over effects from Arabic in their processing of English gender-neutral language. To do so, we explore the following question:

4. Do English monolinguals and Egyptian bilinguals differ in their gender expectations and reliance on stereotypical information? If yes, do Egyptian Arabic/English bilinguals show effects of transferring their knowledge of Arabic grammatical gender to English?

## Theoretical framework

In this section we outline the necessary background information required to contextualise the results, understand the importance and use of this research, as well as our epistemological approach to experimental design.

### How does Egyptian Arabic do gender?

Egyptian Arabic, a diglossic variation of Modern Standard Arabic (MSA), follows the same grammatical gender system as MSA. For the purpose of this experiment, there are no distinctions made between these two variants as role nouns and occupations are consistent across both.

Arabic follows a two-gender system with masculine [+masc] and feminine [+fem] distinctions, and importantly does not possess a ’neuter’ gender option. Similar to Indo-European languages with such a two-gender system (e.g., Spanish), the generic, unmarked, or default form which can be grammatically used to refer to women and men is the masculine. This masculine generic form is typically marked with no overt gender morphology. The feminine form, which is derived from the masculine, is syntactically marked using various morphemes, typically ‘*a(t)’* or ‘*a(h)’*. For animates (humans and various animals), gender assignment is based on semantic information (this information is often the biological sex of the noun) [12]. An example of this can be seen in (1) and (2) below where the ’ *a(h)*’ morpheme is used to transform the masculine noun into feminine. Importantly, this same relationship is also extended to the plural versions of these nouns. It is worth noting that plural masculine generics, relative to their singular counterparts, may be easier to interpret as a mixed-gender group. Despite being more overtly marked, the use of masculine plurals to refer to women in speech is not uncommon (e.g., *nurses* [+masc] to refer to a group of women), whereas the singular masculine would be uncommon in speech.

(1) مدرس
mudares
teacher.M.SG
’teacher[+masc]’
(2) مدرسة
mudares-**ah**
teacher.F.SG
’teacher[+fem]’

While this nominal agreement pattern is similar to many Indo-European languages, unlike Spanish, French, German, etc., Arabic requires gender and number agreement across pronouns, verbs, nouns and adjectives at the inflectional phrase level [12]. Consequently, gender marking is more salient and reinforced in Arabic than Spanish or German. Consequently, adopting gender-inclusive language in Arabic can be more costly and effortful as it would require a large amount of repetition to avoid the use of masculine generics.

### Definitions

In this section we operationalise the definitions and terms to be used throughout this study. Firstly, a key distinction is made throughout this study between grammatical gender (G-gender) and social gender (S-gender). G-gender explicitly refers to the morphology, whereas S-gender indicates a referent, person, or occupational gender expectation. For example, using the masculine generic to refer to a group of *nurses* in Arabic will have masculine G-gender, but social expectations may (or may not) result in a different S-gender interpretation. Importantly, where G-gender information is insufficient or absent, reliance on S-gender information may be utilised to determine the gender of a referent. Additionally, G-gender and S-gender information may be congruent/incongruent, which may induce processing benefits or costs. For example, it may be easier to interpret *engineer* [+ masc] as a man and *nurse* [+fem] as a woman than the other way around. This is because S-gender is typically determined by an individual’s expectations or stereotypical information and is likely to vary due to different socio-cultural backgrounds and personal experiences.

Secondly, when describing a ’male-bias’ in this study, this refers to an over-representation of male referents compared to female referents. Specifically, occurrences of *strict* male interpretations of masculine G-gender, which in principle refers to all S-genders will be considered a male-bias in perception/interpretation. This is to be distinguished from cultural and social biases or attitudes individuals may hold that cannot be attributed to G-gender. Additionally, this male-bias can manifest itself in processing benefits or costs due to violations/confirmations of S-gender expectations (e.g., processing benefits experienced when G-gender is congruent with S-gender (as in *doctor[+masc]* and *nurse[+fem]*) and processing costs when G-gender is incongruent with S-gender (i.e., *doctor[+fem]* and *nurse[+masc]*).

Thirdly, in this study, the term ’number’ always refers to grammatical number (e.g., singular, dual, or plural) unless otherwise stated.

A final note on social gender: during this study binary interpretations of S-gender are more common given the sociocultural background in Egypt, where nonbinary identities are unrecognised and marginalised in formal and informal contexts.

### Speech production

There is consensus in the existing literature that speech production is prefaced by a series of cognitive processes which include different stages relating to the conceptual sphere and preparation, lexical processing, and lemma selection (this includes syntactic categories such as G-gender and number information), and finally articulation and retrieval of phonological components [13, 14]. However, cross-linguistically the order of these processes remains in question [15], especially in inflectional and grammatically gendered languages such as Arabic.

Focusing on lexical and morphemic processing, studies on morphemic decomposition in Arabic show a reliance on root morphemes and not word pattern morphemes (which include gender, tense, number) in processing and organising lexical information [16]. This would suggest that grammatical gender information is secondary to the root information in lexical processing and selection. Based on these findings, we would expect that interpreting semantic information in the generic form would, like other unmarked languages such as English, conform to interpretations that are congruent with social expectations and stereotype associations. For example, *nurse* would be more likely interpreted as a woman and *doctor* as man. However, what is repeatedly shown in other languages, is that masculine grammatical information is almost always likely to reinforce the masculine to male association and promote the retrieval of male exemplars. Importantly, this relationship is found to hold at the bare noun (BN) level where studies on gender priming effects in Italian, German, and Dutch all show that BNs themselves contain gender information, regardless of gender agreement [17, 18]. These findings have major implications for how we model processing of grammatical gender information especially since previous models, such as the WEAVER ++ model, predict that gender information would only be accessed for the sake of agreement at a later stage in processing [8, 19]. Overall, this context is interesting to explore in Arabic as it offers additional information as to how speakers process grammatical gender.

### Epistemological approach

This study is largely influenced by a poststructuralist and postmodernist narrative, whereby language is not a neutral code, but an active influencer of social organisation [20]. It is broadly linked to the Sapir-Whorf hypothesis on linguistic relativity and determinism, which proposes that linguistic constructs shape our understanding of and interactions with the world [21, 22]. However, it does not set out to explicitly test this hypothesis, as the influence of language on cognition is difficult to distinguish from that of other factors which may have confounding effects on cognition. Instead, this study is designed to deepen and broaden our understanding of the perceptual effects of gender-marked language.

## Materials and methods

This report follows the guidance outlined by Van’t Veer and Giner-Sorolla, Bosnjak *et al*. on preregistration in social psychology [23, 24], and the American Psychological Association’s reporting guidelines for experimental studies (PRP-QUANT).

This study adds a novel experimental method to the existing literature on grammatically gendered languages, processing and perception, as well as speech production. Typically, the existing literature has focused on sentence-evaluation paradigms [see 3] and linguistic expression of gender paradigms [see 25]. In this study, we borrow approaches from speech perception and speech production paradigms to explore the way G-gender is processed and produced in Arabic.

### What is the task?

The experiment examines the effect of grammatical-gender on occupational associations through prompted speech production. This is achieved through a speech production task based on an adaptation of the popular board game ’Taboo’ [26]. The participant’s target is to produce as many words as possible related to the prompt to increase their score, without repeating the prompt word itself. Participants are asked to complete this speech production task with a pre-specified time limit, while repeatedly pressing the spacebar, which will act as an attentional load. Working memory and attention is limited [27, 28]. The attentional load in the task can affect the participant’s speech in various ways [29, 30]. In this experiment, the purpose of the attentional load is to reduce speech planning capabilities, thereby increasing reliance on subconscious associations corresponding to implicit beliefs relating to S-gender and occupation.

Participants will be prompted both audibly and visually, where the target word is initially played and visually remains on the screen until the timer runs out. The auditory prompt is to ensure the correct interpretation of the visual target, while avoiding adding diacritics to the visual primes which could result in confounding effects and slower processing for less ‘obvious’ phonetic interpretations [31]. Un-diacretised words presented on their own or out of context can often be ambiguous between several pronunciations. Context is usually sufficient to disambiguate, and therefore outside of pedagogical contexts, fully diacretised text is highly unusual. The visual prompt will be presented on screen for a duration of 20 seconds.

### Design

The experiment employs both between and within variation and is randomised through a block design (5 blocks). The first factor, which varies between participants, is the language condition. There are four main language conditions, and one sub-condition: singular masculine generics, singular feminine g- gender, gender inclusive binomial word-pairs, English gender neutral, and plural masculine generics as the sub-condition (see Table 1).

**Table 1:** Between variation and breakdown of conditions.

Condition		Example
**1.**	Singular generic masculine	مدرس ‘Teacher[+masc]’
**1.1**	Plural generic masculine	مدرسين ‘Teachers[+masc]’
**2**	Singular feminine	مدرسة ‘Teacher[+fem]’
**3**	Gender-inclusive: Feminine/Masculine word-pair	مدرسة/ مدرس ‘Teacher[+fem]/Teacher[+masc]’
**4**	Gender-neutral: English neutral	Teacher

There is one factor that varies within-participant and that is the ‘stereotypicality’ of the role. Role nouns can be associated with men (e.g., *manager*), associated with women (e.g., *nurse*), or neutral (e.g., *student*). All participants are presented with the same 12 role nouns (4 in each stereotype) but in different language conditions.

### Conditions

**Fig 1:**
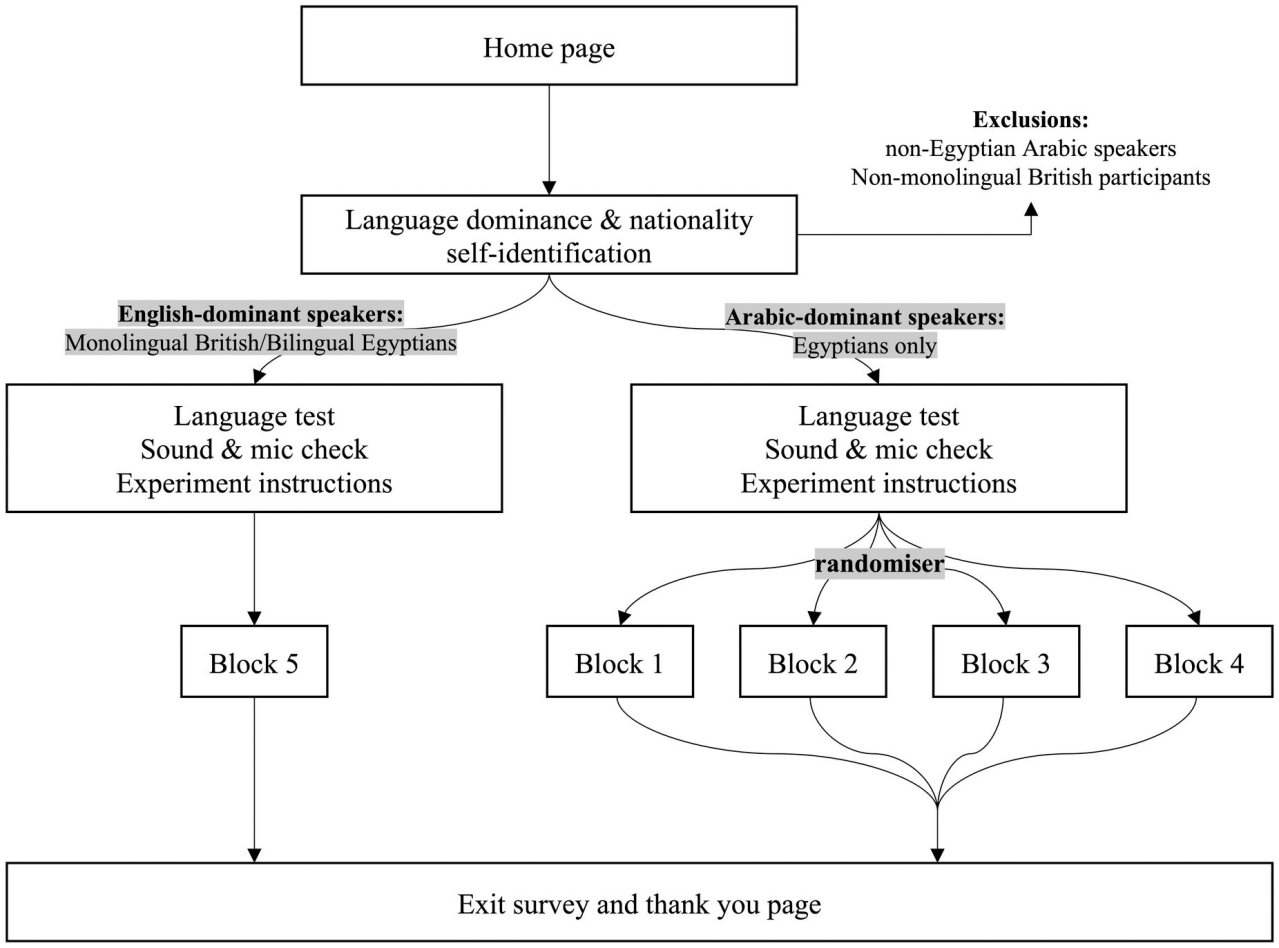
Condition allocation based on participants’ characteristics.

This study can be broken down into three components that help explain experimental blocks and condition allocation in the proposed design. For a detailed breakdown of how participants will be allocated to different blocks based on their characteristics, see Fig 1.

The first component evaluates the effect of Arabic grammatical gender (in the singular) on occupational expectations amongst Egyptian Arabic speakers. In this component, participants are randomly allocated across blocks 1–3 where they are presented with role nouns that vary by language and stereotype condition through a Latin square (see Table 2). Based on this design, 3 different participants are required to complete an observation for one role noun (e.g., *nurse*) in the masculine, feminine, and word-pair conditions. There are several reasons for this choice; firstly, it reduces the chance that participants develop an intuition about the real purpose of the experiment. Secondly, it prevents participants from anticipating the G-gender of the stimuli. Thirdly, this design helps avoid responses that are strictly based on priming patterns and local syntactic consistency [32].

**Table 2:** Latin square blocks for the singular Arabic conditions.

Block	Male Stereotype	Female Stereotype	No Stereotype
**Block 1**	مدير Manager [+ masc]	ممرضة Nurse [+ fem]	تلميذة/تلميذ Student [+ fem/masc]
	مهندسة/مهندس Engineer [+ fem/masc]	راقص Dancer [+ masc]	مغنية Singer [+ fem]
	محامية Lawyer [+ fem]	مضيفة/مضيف طيران Flight Attendant [+ fem/masc]	ممثل Actor [+ masc]
	دكتور Doctor [+ masc]	عاملة نظافة Cleaner [+ fem]	مسؤولة / مسؤول مبيعات Salesperson [+ fem/masc]
**Block 2**	مديرة/مدير Manager [+ fem/masc]	ممرض Nurse [+ masc]	تلميذة Student [+ fem]
	مهندسة Engineer [+ fem]	راقصة/راقص Dancer [+ fem/masc]	مغني Singer [+ masc]
	محامي Lawyer [+ masc]	مضيفة طيران Flight Attendant [+ fem]	ممثلة/ممثل Actor [+ fem/masc]
	دكتورة / دكتور Doctor [+ fem/masc]	عامل نظافة Cleaner [+masc]	مسؤولة مبيعات Salesperson [+ fem]
**Block 3**	مديرة Manager [+ fem]	ممرضة/ممرض Nurse [+ fem/masc]	تلميذ Student [+ masc]
	مهندس Engineer [+ masc]	راقصة Dancer [+ fem]	مغنية/مغني Singer [+ fem/masc]
	محامية/محامي Lawyer [+ fem/masc]	مضيف طيران Flight Attendant [+ masc]	ممثلة Actor [+ fem]
	دكتورة Doctor [+ fem]	عاملة/عامل نظافة Cleaner [+ fem/masc]	مسؤول مبيعات Salesperson [+ masc]

The second component is considered an exploratory sub-experiment of the first which evaluates the effect of plural masculine generics on the occupational expectations of Egyptian Arabic speakers. The key difference between this component and the former is that this block does not test this relationship in other language conditions (such as feminine plurals or in binomial word-pairs) which would require 3 additional blocks instead of one. This component will be used to explore one key question: do plural masculine generics and singular masculine generics elicit the same male-bias in representation? Importantly, this exploratory sub-experiment has 6 experimental stimuli (in the masculine) compared to the other blocks (see Table 3). This is to ensure a balance of masculine and feminine priming throughout the experiment.

**Table 3:** Experimental block for the plural masculines.

Block	Male Stereotype	Female Stereotype	No Stereotype
**Block 4**	**مديرين** **Managers [+ masc]**	راقصة Dancer [+ fem]	تلميذة/تلميذ Student [+ fem/masc]
	محامية/محامي Lawyer [+ fem/masc]	**ممرضين** **Nurses [+ masc]**	مسؤولة مبيعات Salesperson [+ fem]
	دكتورة Doctor [+ fem]	**مضيفين طيران** **Flight Attendants [+ masc]**	**ممثلين** **Actors [+ masc]**
	**مهندسين** **Engineers [+ masc]**	عاملة نظافة Cleaner [+ fem]	**مغنيين** **Singers [+ masc]**

The third component explores the effect of an absence of grammatical gender on occupational expectations in a gender-neutral language (English). In this component Egyptian bilinguals and British English monolinguals will complete the experimental task with English stimuli (see Table 4). This component will also be used to explore differences between the occupational expectations of English monolinguals and Egyptian bilinguals. The presence of the English monolingual participants acts as a ‘control group’ for the experiment as they cannot be influenced by G-gender information. All their responses should be driven by their stereotypical expectations of the role nouns presented. On the other hand, the bilingual Arabic/English speaker is expected to behave differently. Bilinguals have been shown to shift their perception of gender by language [8, 33]. In the absence of grammatical gender information, the design may allow us to directly access participants occupational expectations based on social gender. However, participants may also showcase gender ‘carryover effects’ from Arabic grammatical gender instead of relying on social stereotypes. This could be seen in instances where participants consistently describe the referent using masculine morphosyntax (e.g., ‘he’, ‘him’, ‘his’), even when the role stereotype is associated with women (e.g., describing a male nurse, secretary, or dancer), due to the Arabic masculine generic convention.

**Table 4:** Experimental block for English gender-neutral condition.

Block	Male Stereotype	Female Stereotype	No Stereotype
**Block 5**	Manager	Nurse	Student
	Engineer	Dancer	Singer
	Lawyer	Cleaner	Salesperson
	Doctor	Flight Attendant	Actor

### Outcomes of interest

To answer the research questions outlined above, the study focuses on two outcome categories: fluency measures and the participant’s gender interpretation of the prime.

Fluency measures consist of 5 dependent variables: the total number of words produced, hesitation markers, number of false starts, fluency mistakes, and initial response time. These measures are consistent with current literature on speech production [34]. The focus on fluency allows for exploration of processing costs and benefits due to in/congruent or asymmetric stimuli. Additionally, fluency measures help show what is easily accessed or recalled. Processing benefits are expected when participants’ S-gender expectations are not violated (i.e., a *secretary* in feminine morphology and a *manager* in masculine morphology compared to a *secretary* in the masculine). Importantly however, roles associated with women presented in masculine morphology should *not* result in processing costs if masculine G-gender is truly interpreted as generic or unmarked.

To measure the participant’s interpretation of the prime their responses will be coded based on the proportion of gendered morphosyntactic markers in their speech. Importantly, this will be weighted at the tensed phrase (TP) level. This is to avoid inflating scores based on morphosyntactic markers for the sake of agreement across noun phrases or verb phrases. This coding will be used to derive two other categorical variables to be used in the analysis: the participant’s gender expectations and whether the expectations are congruent with the social stereotype associated with the prime.

Example 3 provides a fictitious response to the stimulus “*manager [+masc]*” and how this would be coded. Firstly, we can see that there are three morphosyntactic markers that are masculine, two of which are used only for agreement purposes. Secondly, we can determine that the participant interpreted the stimulus to be a man and that this response is congruent with the prime (i.e., grammatical gender and social gender are not asymmetric).

**Table:** 

(3) هو بيكون المسؤول		
huwa	biko:n	mas2o:l
he.GEN.**M**.3SG	3PROG.**M**.is	DEF.responsible.**M**.SG.ADJ
‘He is [+masc] responsible [+masc]’		

### Hypotheses

As seen in Table 5 predictions for the directional relationships of the independent and dependent variables are made. These predictions are described and motivated in greater detail below.

**Table 5:** Directional relationships between independent and dependent variables.

VARIATION			OUTCOMES						
between	within		gender interpretation		fluency				
condition	association and number		referent expectations	proportion of gendered morphosyntactic markers	no of words produced	fluency mistakes	number of false starts	hesitation markers	initial response time
1. Generic masculine	man	SG	man	high proportion of masculine morphosyntactic markers	high	low	low	low	low
		PL							
	woman (incongruent)				low	high	high	high	high
	neutral				high	low	low	low	low
2. Feminine G-gender	man (incongruent)		woman	high proportion of feminine morphosyntactic markers	low	high	high	high	high
	woman				high	low	low	low	low
	neutral				high	low	low	low	low
3. Gender fair language (fem/masc)	man		interpretations can be a) mixed group b) congruent with stereotype	presence of both feminine and masculine morphosyntactic markers with higher proportion of masculine	no significant differences for stereotype or number variation				
	woman								
	neutral								
4. English gender neutral	man		man	presence of feminine, masculine, or neutral pronouns depending on stereotype	no significant differences for stereotype or number variation				
	woman		woman						
	neutral		unknown						

For conditions with strict masculine or strict feminine G-gender: regardless of role stereo-typicality participants’ responses will be determined by the G-gender of the prime. In other words, the priming effect of G-gender will overpower the priming effect of social stereotype information and participants will strictly rely on G-gender rather than stereotype information to determine the S-gender of the referent. Further, this relationship will be more extreme in the singular compared to the plural (as the singular is less likely to be interpreted as generic compared to the plural). Consequently, it is expected that masculine generics will result in a male bias in perception and interpretation. Similarly, feminine G-gender **should** also result in female interpretation as this cannot be used generically. Importantly, if the masculine and feminine conditions behave similarly, this will confirm the notion that masculine generics are not actually generic in interpretation and are instead gender exclusive.

Further, it is expected that the neutral roles in the masculine generic condition will be more readily interpreted as stereotypically male and may also act as a ‘congruent’ condition. Therefore, participants’ responses towards ‘neutral’ roles (such as *student*) will mirror responses towards male-dominated roles.

In condition 3 (inclusive language), it is unclear how participants will interpret the stimuli. Firstly, due to the presence of gender-fair language, we would expect participants to use both feminine and masculine morpho-syntactic markers across all roles, with the neutral stereotype condition being the most compatible with this behaviour. In this scenario, participants will have shown that gender inclusive language can be used to overcome male bias in perception and increase the retrieval of female exemplars. Alternatively, participants’ gender expectations and interpretation of the prime may instead be determined by social stereotype information. In this case, we can determine that despite the presence of a feminine morphosyntactic marker in the stimulus and an increase in accessibility of female exemplars, gender inclusive language does not increase the retrieval of female exemplars in the presence of competing social stereotype information (i.e., a failure to overcome gender bias in perception because of social expectation).

Similarly, in condition 4 (neutral English), stereotypical information may inform participants’ gender expectations, such that *doctors* are more likely to be men and *secretaries* are more likely to be women in responses. If this is the case, then the fixed effect for language in conditions 3 and 4 should not be statistically different. Importantly, in this condition participants have the option to respond in the gender neutral ‘they’ instead of assuming the role’s gender. Therefore, some variation between feminine, masculine, and neutral pronouns could be expected. If this condition also elicits highly gendered responses based on social stereotypes, then this presents evidence on the importance of gender-fair and representative language rather than just neutral language. This has implications for jobs advertised in English, which are assumed to be gender-neutral in interpretation but may instead entrench participant’s existing stereotypes.

Additionally, it is expected that fluency measures in congruent conditions will exhibit processing benefits (i.e., fewer fluency mistakes, more speech produced within the time limit, less hesitation, and reduced lag time) compared to their incongruent counterparts. Where grammatical information and stereotype information are incongruent (i.e., *nurse* presented in the masculine or *manager* presented in the feminine), it is predicted that outcomes related to fluency and initial response time will be negatively affected and participants will experience processing costs as a result of a violation of expectations. Importantly, interpreting masculine G-gender as generic should not be as costly or inefficient because it can be used to refer to male or female S-genders.

Further, condition 3 should not show significant differences in fluency or response time due to stereotype or number variation. This is because G-gender and stereotype information can always be congruent in a word-pair. Similarly in condition 4, it is predicted that fluency and response time will not vary across stereotype or number.

It is worth noting that condition 3 (inclusive language) as a whole compared to other conditions may trigger longer response times and processing costs because the word-pair may lead to initial hesitation as to which form of gender agreement the participant should select given that both the masculine and feminine are presented. Additionally, fem/masc combinations are not as frequent as masc/fem combinations and so some order effects/lag time are expected in this condition. This condition may also elicit plural morpho-syntactic markers if participants interpret the word pair as having both one male and one female referent.

Similarly, condition 2 (feminine G-gender) as a whole is likely to show processing costs, as role nouns associated with men or those that are neutral are lower frequency in the feminine compared to their masculine counterparts. That being said, both the feminine and masculine versions of a role noun share identical root morphemes and vary only in suffixes so differences in fluency can directly be attributed to the infrequent use of feminine role nouns. Consequently, the English version of the experiment, and masculine generic conditions in Arabic are expected to trigger higher fluency scores compared to the other conditions.

#### Participants’ characteristics

Participants’ age, gender, stereotype beliefs, or level of aversion to women in the workplace will be collected to check for mediating effects on gender expectations and responses to stimuli in the gender-exclusive conditions. Bilingual Egyptian participants and monolingual UK respondents are expected to show differences in the gender-neutral condition. Cultural differences in the perception of women and differences in occupational expectations may affect their responses. Further, bilingual Egyptians might be more likely to interpret English neutral language as masculine due to carryover effects from Arabic as well as a more obvious level of gender segregation in Egypt compared to the UK where some of the gender stereotypes may not be as salient. Crucially, if neutral G-gender primes male occupational expectations for both bilingual and monolingual English speakers, this will show large potential for change through attention to inclusive and gender-fair language in both Arabic and English-speaking contexts. However, if significant differences arise between both types of participants this could help show the influence of either cultural differences (as proxied by the exit survey on aversion to women in the workplace) or linguistic differences and gender carryover effects from Arabic to English. The current design of the experiment is incapable of fully disambiguating these cultural and linguistic factors, but this could point to an important area of future research on bilingual processing to determine if carryover effects are driving this variation or sociocultural differences.

### Sampling & participant recruitment

#### Sample characteristics

There are three types of participants that will be recruited for the study: ‘native’ Egyptian Arabic speakers, monolingual British English speakers, and Egyptian bilinguals with ‘native level’ proficiency in both English and Arabic. Fig 1 presents the possible blocks that different participants can be allocated to depending on their characteristics. For all Arabic blocks, Arabic speakers residing in or who have previously resided in Egypt for at least 10 years will be recruited. All other Arabic-speaking countries will be excluded, this is largely because the norming and stereo-typicality of the stimuli is based on an Egyptian sample and so the ecological validity of these categories has not been tested in other Arabic-speaking settings. However, this is not expected to affect the generalisability of the results as the classifications of these roles are likely to be regionally consistent.

Regardless of language spoken, the study will be targeting a gender-balanced sample of participants aged 18 and above. There will be no restrictions placed on age, but age will be included in the mixed effects model as a potential moderating factor.

#### Participant recruitment & data collection

Both the English-speaking and Arabic-speaking sample will be recruited through online resources (Twitter, Facebook, and Linked-In), university mailing lists, and Prolific (if needed). Data will be collected online (on the participants’ personal device) and in person (in a university lab).

#### Exclusion criteria

Participants not sufficiently proficient in English (for the English Condition) or Arabic (for the Arabic conditions) will be excluded from the analysis. Proficiency will be determined using a C-test, and results below native-level proficiency will be excluded (this corresponds to a score of less than 90% on the test). Additionally, participants that are aware of the purpose of the experiment, based on their responses in the exit survey, will be excluded. Finally, participants that repeatedly or purposely ignore the two key tasks (pressing spacebar and producing speech) for more than 2 occupations will be excluded.

#### Sample size

As this is a novel experiment, an estimation of the effect size based on the previous literature or pilot data is not possible. Consequently, a power analysis cannot be conducted at this stage. As such, the study will initially target the average sample size (30 participants) per condition in the relevant existing literature. Therefore, at present the sample will consist of at least 30 participants in blocks 1–4, and 30 Egyptian bilinguals plus 30 monolinguals in the English block (total of 180). However, after conducting the pilot we will use the results to conduct a formal power analysis in STATA (power 0.9 and *alpha* 0.1) to check if additional participants are required.

### Ethics

This study has been reviewed and approved by the Queen Mary Ethics of Research Committee on the 9^th^ of February 2022 and the approval is valid for 3 years from the date of approval–Reference Number: QMERC20.601. In accordance with the Queen Mary Ethics of Research Committee, written informed consent will be obtained from participants online.

## Procedure

Firstly, participants will be asked to self-identify themselves as proficient in either Arabic, English or both. Participants selecting just Arabic or English will be asked to complete a C-test in their selected language. Participants selecting both English and Arabic (i.e., bilinguals) will be asked to complete a C-test in both languages. Upon passing the language tests (and audio/mic checks), participants will be randomly allocated through to the blocks they are eligible for (see Table 6). Both the participants and the researcher will be blinded to their block allocation.

**Table 6:** Expected timeline.

Phase	Activity	Expected completion date
1	Norming stimuli and online experiment design	15 June 2023
2	Piloting	15 July 2023
3	Participant recruitment and data collection	October–December 2023
4	Data cleaning and analysis	January 2024
5	Writing and submission	January–March 2024

Once allocated to their blocks, participants will be presented with instructions and a consent form. They will be informed that the task is about multitasking and memory and that to increase their score, they are required to produce as much speech as possible while simultaneously and repeatedly pressing a designated button. As this is modelled after a game, participants are incentivised to produce more words to increase their score. Next, participants will be given 3 practice stimuli to familiarise themselves with the setup and avoid any order effects.

After completing the experiment, participants will be presented with an exit survey. The exit survey will collect participants’ perception of the experiment’s purpose, demographic details (including age, years of education, gender, years of employment), attitudes towards women in the workplace/aversion to women in the workplace, explicit attitudes towards gender-marked language in recruitment, language dominance measures, and number of languages spoken. Finally, participants conducting the experiment will be paid EGP 250 or GBP 6.5 depending on country of residence. The session is expected to last for 30 minutes (including the exit survey and practice rounds).

## Materials

Experimental stimuli and role nouns have been selected based on a corpus collected from online Egyptian recruitment websites [11] and a large scale norming study on occupational stereotypes [35]. These roles were then cross-checked on Sketch Engine to ensure that none of the roles selected are low frequency. For an occupation to qualify as stereotypically female, it has to be typically advertised to women and advertised in feminine grammatical gender (such as *nurse* and *secretary*). Similarly, these roles on Sketch Engine were more likely to appear in feminine G-gender compared to their masculine counterpart, which further validates the stereotypicality of the role. For stereotypically male roles, the same approach was adopted, and occupations were selected if they mostly (at least 70%) targeted men and used masculine grammatical gender (such as *lawyer* and *manager*).

There were two main challenges when selecting the experimental stimuli. Firstly, the gendered stereotypes need to hold cross-culturally for both Egyptian and British participants. Secondly, as the existing corpus and literature has shown, Egyptian online recruitment is heavily gender segregated and polarised. Consequently, finding ‘neutral’ roles in the corpus proved to be difficult and therefore 3 of the neutral stimuli were selected based on other norming studies’ classifications [3, 35].

To mitigate these challenges and ensure that the stereotypes match the attitudes of the target sample(s), additional norming during the piloting stage will be conducted. This will consist of a simple questionnaire to be circulated amongst an Egyptian and British sample (n = 10 for each) asking participants to rate the occupations selected (this includes 4 plus an additional 2 ‘back-up’ role nouns in each stereotype) as stereotypically male, female, or neither on a 7-point scale. Role nouns with stereotypes that do not hold cross-culturally will be excluded from the experiment, with the target of having 4 occupations for each stereotype.

Non-experimental stimuli will include animals (such as *cat*) and will be G-gender balanced with 2 nouns presented in feminine G-gender and 2 presented in masculine G-gender.

## Piloting

This experiment will be piloted in one round if there are no adjustments necessary based on the piloting. However, if changes are required based on the initial pilot, an additional smaller round of piloting will be conducted, and size will be determined based on the magnitude of the change. For example, if the change only affects block 5, other blocks will not be subject to additional piloting. In the first round, blocks 1–3 will be piloted with 3 participants each, block 4 will be piloted with 4 participants, and block 5 will be piloted with 6 participants (3 Egyptian bilinguals and 3 English monolinguals). Based on the initial pilot, the following changes can be made. Firstly, stimuli will be changed (if necessary) based on the stereotype norming exercise. Secondly, the time allocated to participants to produce speech can be increased or decreased from the initial 20 seconds if this is found to be insufficient or too abundant. If no changes are made based on the pilot, the participants’ observations will be included in the analysis.

## Pre-specified analysis plan

The study uses mixed effects regression, modelled in STATA, to account for within and between variation in the experiment. There are two different outcome categories and therefore multiple models will be used to explore the effect of language on the dependent variables outlined above.

The first set of models related to fluency will utilise linear-mixed-effects models, and models related to gender expectations will use generalised linear mixed effects models for categorical dependent variables. In both cases, the language condition will be defined as the fixed effect. The models will include random slopes for this fixed effect as some occupations may be more affected by the language condition than others. By-participant random slopes and intercepts will be included in the models as random effects. This is because participant variation is expected due to differences in demographic information (age and gender), attitudes towards women (from the exit survey), language dominance measures (exit survey), and participant’s nationality (note that this is likely corelated with attitudes towards women, and highly correlated with language dominance measures). Similarly, by-item random slopes and intercepts will be included as variation due to length of stimuli or familiarity with different occupations should be accounted for.

Interaction effects will be explored between stereotype and language condition, and for a subsample of the data interactions between number and stereotype will also be explored. Interaction effects related to number will help assess the mediating effect of number on gender expectations in masculine grammatical gender.

### Data processing & cleaning

In this section we describe how we handle outliers, missing data, and model specification. Firstly, given the presence of by-item and by-participant slopes and intercepts, outliers are less likely to be problematic for the analysis. However, as an additional check, using the *extremes* command on STATA for all numeric outcomes (i.e., fluency measures) we will initially check for cases with extremely high or low values. If any extreme values are identified, they will be investigated further by checking their residuals and leverage. A sensitivity analysis will be conducted to determine whether outlier data will be included or excluded in the analysis. If results are heavily affected by the inclusion of an outlier (and there is no evidence to suggest that differences in results are not random) then this data will be excluded from the analysis.

Secondly, if there is more than 5% of the data missing, this will be further investigated to check if by-item attrition is systematic or randomly distributed. If it is randomly distributed, then these missing observations are automatically removed by STATA in analysis. However, if the missing observations are systematic (e.g., most of the missing data is in response to one particular occupation or stimuli) then this will be investigated further and potentially dropped from the analysis.

Finally, to avoid an overspecification of the models, models that include interactions as predictors will be compared to a simplified model that excludes these interactions using the Bayesian Information Criterion (BIC). The BIC will be used to determine if the unrestricted or restricted model is a better fit for the data.

## Timeline

Table 6.
